# Effect of the ketogenic diet on glycemic control, insulin resistance, and lipid metabolism in patients with T2DM: a systematic review and meta-analysis

**DOI:** 10.1038/s41387-020-00142-z

**Published:** 2020-11-30

**Authors:** Xiaojie Yuan, Jiping Wang, Shuo Yang, Mei Gao, Lingxia Cao, Xumei Li, Dongxu Hong, Suyan Tian, Chenglin Sun

**Affiliations:** 1 Department of Clinical Nutrition, First Hospital of Jilin University, 1 Xinmin Street, 130021 Changchun, Jilin China; 2 Department of Endocrinology and Metabolism, First Hospital of Jilin University, 1 Xinmin Street, 130021 Changchun, Jilin China; 3 Division of Clinical Research, First Hospital of Jilin University, 1 Xinmin Street, 130021 Changchun, Jilin China

**Keywords:** Type 2 diabetes, Nutrition

## Abstract

**Background:**

At present, the beneficial effect of the ketogenic diet (KD) on weight loss in obese patients is generally recognized. However, a systematic research on the role of KD in the improvement of glycemic and lipid metabolism of patients with diabetes is still found scarce.

**Methods:**

This meta-study employed the meta-analysis model of random effects or of fixed effects to analyze the average difference before and after KD and the corresponding 95% CI, thereby evaluating the effect of KD on T2DM.

**Results:**

After KD intervention, in terms of glycemic control, the level of fasting blood glucose decreased by 1.29 mmol/L (95% CI: −1.78 to −0.79) on average, and glycated hemoglobin A1c by 1.07 (95% CI: −1.37 to −0.78). As for lipid metabolism, triglyceride was decreased by 0.72 (95% CI: −1.01 to −0.43) on average, total cholesterol by 0.33 (95% CI: −0.66 to −0.01), and low-density lipoprotein by 0.05 (95% CI: −0.25 to −0.15); yet, high-density lipoprotein increased by 0.14 (95% CI: 0.03−0.25). In addition, patients’ weight decreased by 8.66 (95% CI: −11.40 to −5.92), waist circumference by 9.17 (95% CI: −10.67 to −7.66), and BMI by 3.13 (95% CI: −3.31 to −2.95).

**Conclusion:**

KD not only has a therapeutic effect on glycemic and lipid control among patients with T2DM but also significantly contributes to their weight loss.

## Introduction

Diabetes mellitus (DM) is the world’s leading cause for motility and morbidity, and the disease has become a major public health burden worldwide. It is estimated that the prevalence of diabetes in adults worldwide is over 300 million, and it will increase by 55% by 2035^1^. Obesity or overweight is one of the essential risk factors for diabetes and contributes to a twice-higher risk to develop DM^2,3^. Thus, dietary therapy aiming at weight loss is typically recommended in clinical practice^4^. Due to the fact that diabetes and its complications affect many aspects of physiology, the benefits of weight reduction are not limited to glycemic control but are also related to many cardiovascular risk factors such as blood pressure, high-density lipoprotein (HDL), total cholesterol (TC) and triglyceride (TG)^2^.

Medical nutrition, as part of the comprehensive treatment of DM with obesity with a primary goal of weight reduction, is the most simple, effective and economical choice of intervention. The dietary approach for body weight reduction can be obtained from many strategies, including a low-calorie diet, a very low-calorie diet, high-protein diet, and so on. Ketogenic diet (KD), which contains a very low level of carbohydrates (<55 g/d) with the main energy sources of lipid and protein, and which causes ketosis and simulates the physiological state of fasting, has been well reported to be effective for weight loss and glycemic control^4–9^. Previous meta-analyses have proved the efficacy of KD in body weight reduction^2,10,11^; however, systemic reviews on the effect of KD on weight reduction and glycolipid metabolism in patients with DM are still limited. Westman et al.^12^ and Partsalaki et al.^13^ demonstrated that KD improved type 2 diabetes mellitus (T2DM) by reducing the glycemic response caused by carbohydrate and improving potential insulin resistance. Leonetti et al.^14^ and Walton et al.^15^ reported reduced TG and TC with increased HDL levels after KD consumption for a lipid profile. However, controversies are still existing; studies revealed that a low-carbohydrate, high-fat diet may exacerbate the lipid profile in patients with diabetes, although glycemic control improved with hypoglycemic medications^16–18^. Therefore, the purpose of the current review was to conduct a meta-analysis on the effects of a KD in patients with diabetes.

Considering the potential benefits of KD in diabetes management and weight reduction, and considering fasting blood glucose and glycated hemoglobin A1c (HbA1c) as common biomarkers for long-term glycemic control, HDL, LDL, TC, and TG levels are included in the current analysis to determine the changes of metabolic disorders in glucose and lipid metabolism. In addition, the homeostatic model assessment of insulin resistance (HOMA-IR) is considered as a reflection of insulin resistance reversal.

## Materials and methods

### Literature search

**Fig. 1:**
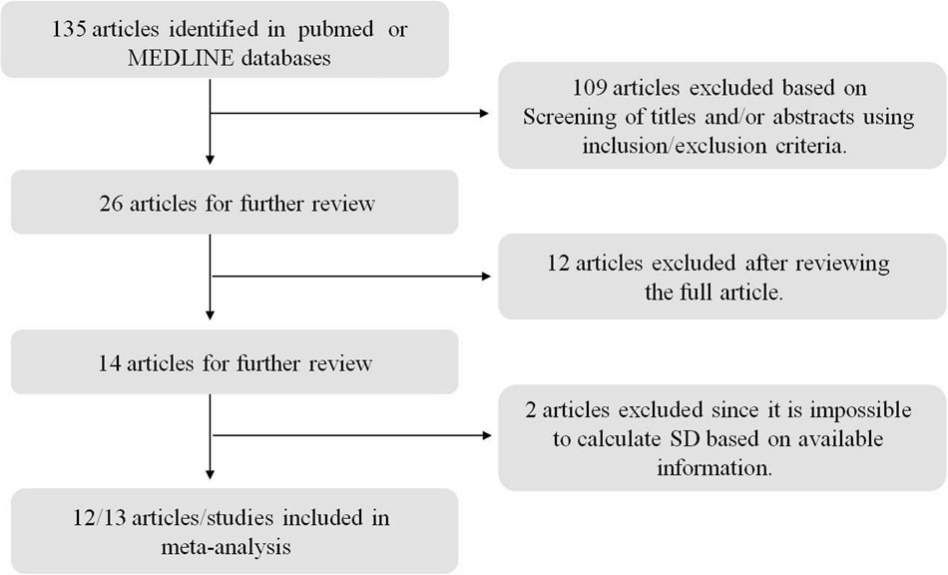
PRISM diagram for the systematic review. Only studies published in English were considered, which were identified by searching the PubMed and MEDLINE databases. The keywords used for this literature search are T2DM or diabetes mellitus, ketogenic diet, obesity, and human. The search was finished on September 20, 2019.

In this meta-analysis, only studies published in English were considered, which were identified by searching the PubMed and MEDLINE databases. The keywords used for this literature search are T2DM or diabetes mellitus, ketogenic diet, obesity, and human. The search was finished on September 20, 2019. This meta-analysis was planned and performed according to the Preferred Reporting Items for Systemic Reviews (PRISM) guideline (Fig. 1).

### Inclusive/exclusive criteria

Studies that met the following inclusive criteria were included: (1) the disease of interest is type II diabetes; (2) the therapeutic diet under consideration is KD; (3) the study was carried out on humans; animal experiments are not included; and (4) the summary statistics of the mean difference between before and after KD (if both means for before and after measurements are available, then we took the difference of these two statistics to obtain the desired mean difference), their corresponding standard error or 95% CI (according to this, the standard error was calculated) or *p* values (according to this, the corresponding *t* statistics and subsequently the standard error were calculated) are available.

Exclusive criteria: (1) case report studies were excluded; (2) meta-analysis or review studies were excluded; (3) studies on other diseases rather than type II diabetes were excluded; and (4) if only the respective mean and standard errors were available, such studies were excluded given it is hard to get an accurate estimation for the standard error of mean difference (since both measurements were on the same patient, they should be correlated to each other, and hence it is impossible to estimate this correlation).

### Statistical analysis

The effects of KD on type II diabetes were estimated by the mean difference after KD versus before KD and their corresponding 95% CIs in random-effects meta-analysis models or fixed-effect meta-analysis models. To determine which model should be used, heterogeneity among studies was evaluated by the Cochrane’s Q statistic corresponding *p* values and the *I*^2^ statistics. If the *p* value was <0.05 and *I*^2^ > 0.5, a random-effect meta-analysis model was used. Otherwise, a fixed-effect meta-analysis model was chosen. Additionally, potential bias was assessed by using funnel plots, in which effect sizes versus standard errors were diagrammed. All statistical analysis was carried out in the R software, version 3.5 (www.r-project.org)^19–21^.

## Results

There are 13 studies included in this meta-analysis; the details of these 13 studies are presented in Table 1. In total, 567 subjects were included in the final meta-analysis. From the perspective of glucose metabolism, lipid metabolism, and weight control, the effects of KD on T2DM were systemically reviewed by comparing the after-intervention measures with before-intervention measures of several biomarkers for the same patient. The variables used to surrogate for carbohydrate metabolism are included fasting glucose level and HbA1c; for lipid metabolism TC, TG, HDL and LDL; and for weight loss body weight, BMI and waist circumference. For all variables except BMI and waist, random-effect models were adopted according to the *Q* statistic *p* value and *I*^2^ statistics.

**Table 1:** A summary of participants’ characteristics of studies included.

	Study	Country	*N*	Follow-up (week)	Dietary intervention	Age	BMI	Diabetes duration	Diabetes assessment
1	Tay^38^	Australia	55	52	Low-carbohydrate (LC) diet: 14% carbohydrate (carbohydrate <50 g/d), 28% protein, 58% fat, <10% saturated fat, 35% monounsaturated fat, 13% polyunsaturated fat.	58 ± 7	34.2 ± 4.5	7 ± 5	Self-report hospital diagnosis
2	Makenzie^22^	USA	238	10	Very low-calorie diets (VLCDs): <30 g/day carbohydrate, 1.5 g/kg protein, incorporate dietary fats to satiety.	54 ± 8	40.8 ± 8.9	Not specified	Self-report
3	Westman^12^	USA	21	24	Low-carbohydrate, ketogenic (LCK) diet: <20 g/day carbohydrate.	51.2 ± 6.1	37.8 ± 6.7	>1	Self-report, hospital diagnosis
4	Yancy^23^	USA	21	16	Low-carbohydrate, ketogenic diet (LCKD): ≤20 g/day carbohydrate.	56 ± 7.9	42.2 ± 5.8	Not specified	Self-report, FPG
5	Dashti^29^	Kuwait	31	56	Low-carbohydrate, ketogenic diet (LCKD): <20 g/day carbohydrates, 80–100 g/day proteins, additional 20 g/day carbohydrates after 12 weeks.	46.4 ± 9.4	≥30	Not specified	Self-report
6	Myette-Cote^43^	Canada	11	1	Low-carbohydrate high-fat diet (LC): 10% carbohydrate, 25% protein, 65% fat.	64 ± 8	34.0 ± 8.0	Not specified	Screening ADA, 1998
7	Myette-Cote (+walk)^43^	Canada	11	1	Low-carbohydrate high-fat diet with 15-min post-meal walks (LC + Ex): 10% carbohydrate, 25% protein, 65% fat,15 min of walking beginning 30 min after breakfast, lunch, and dinner.	64 ± 8	34.0 ± 8.0	Not specified	Screening ADA, 1998
8	Goday^41^	Spain	45	18	Very low-calorie, ketogenic (VLCK) diet: <50 g/day carbohydrate.	54.89 ± 8.81	33.25 ± 1.52	≥10	Self-report
9	Leonetti^14^	Italy	14	4	Very low-calorie ketogenic diet (VLCKD): 15 g/day carbohydrates, 72–80 g/day proteins, 23–24 g/day lipids	47.7 ± 11.2	50.8 ± 6.2	Not specified	Screening ADA, 1998
10	Saslow (2014)^35^	USA	15	36	Low-carbohydrate, ketogenic (LCK) diet: 20–50 g/day carbohydrates.	≥18	36.2 ± 8.2	Not specified	Self-report Diabetes medication
11	Saslow (2017)^9^	USA	16	48	Low-carbohydrate ketogenic (LCK) diet: 20–50 g/day carbohydrates.	≥18	35.9 (32.5, 39.2)	Not specified	Self-report, Diabetes medication
12	Walton^15^	USA	11	13	Low-carbohydrate (LC) ketogenic diet: 5% carbohydrate (carbohydrate < 30 g/day), 20−25% protein, 70−75% fat.	38.3 ± 2.6	36.3 ± 1.4	≤1	Self-report hospital diagnosis
13	Hussain^4^	Kuwait	78	24	Low-carbohydrate ketogenic diet (LCKD): 20 g/day carbohydrate.	37.2 ± 0.4	40.0 ± 0.7	Not specified	Self-report, FPG

*N* number of participants recruited in the study.

**Fig. 2:**
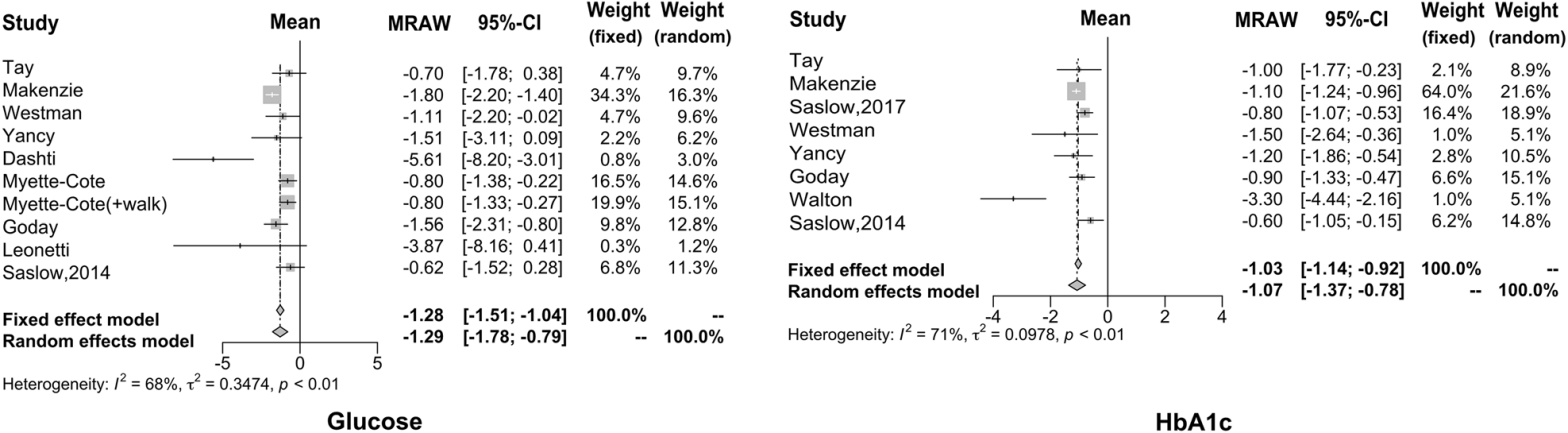
Forest plots for blood glucose and HbA1c. The reduced proportion of HbA1c is more significant after the KD implementation, which is regarded as the ideal therapeutic effect of drugs that is possible to be achieved on HbA1c.

Using the meta-analysis method, we found that the fasting blood glucose level was decreased 1.29 mmol/l (95% CI: −1.78 to −0.79) after the intervention of KD, compared to before such an intervention (based on ten articles that have the summary statistics for the difference between after- and before-intervention measures). As far as HbA1c is concerned, we found that the reduced proportion of HbA1c is more significant after the KD implementation, with a difference of −1.07% (95% CI: −1.37 to −0.78), which is regarded as the ideal therapeutic effect of drugs that is possible to be achieved on HbA1c. The forest plots for these two carbohydrates metabolism indices are given in Fig. 2.

**Fig. 3:**
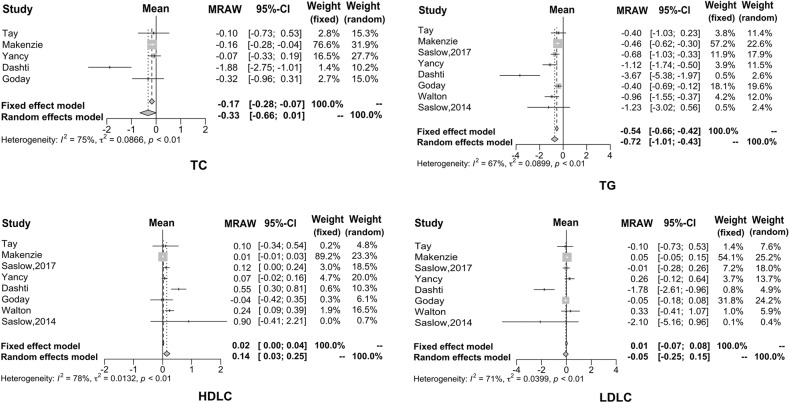
Forest plots for TC, TG, LDL, and HDL. It can be seen that after KD consumption, TG, TC, and LDL decreased. On the other hand, HDL increased.

In this study, eight articles investigated the effect of KD on the lipid metabolism of diabetic patients, but only five papers analyzed total cholesterol. It can be seen that after KD consumption, TG decreased by 0.72 mmol/L (95% CI: −1.01 to −0.43), TC decreased by 0.33 mmol/L (95% CI: −0.66 to −0.01), and LDL decreased by 0.05 mmol/L (95% CI: −0.25 to −0.15). On the other hand, HDL increased by 0.14 mmol/L (95% CI: 0.03−0.25). The forest plots for these four biomarkers are shown in Fig. 3.

**Fig. 4:**
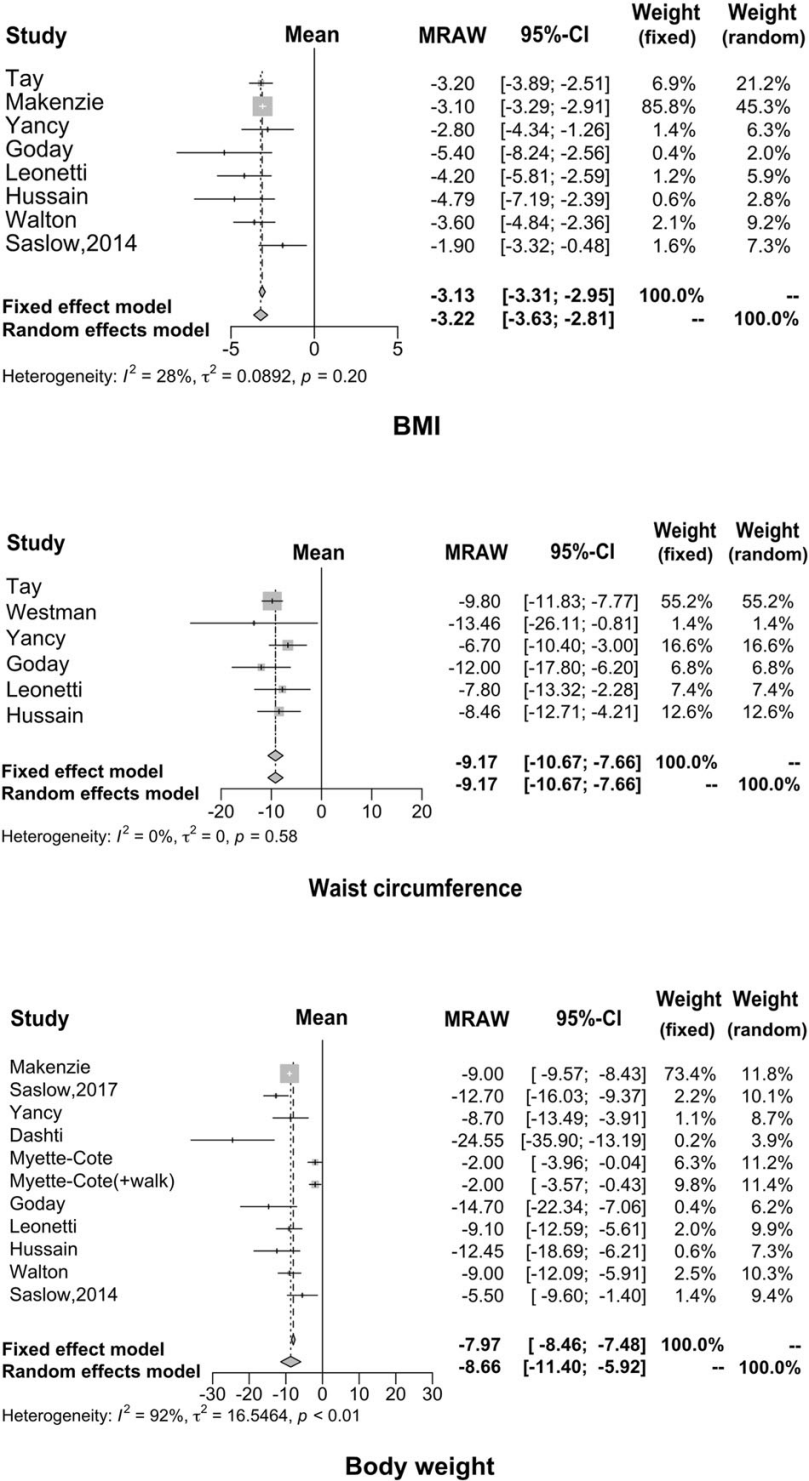
Forest plots for body weight, waist circumference, and BMI. Many studies have demonstrated that KD has a positive effect by providing effective control over obesity; our findings were consistent with the previous reports.

Regarding weight loss, many studies have demonstrated that KD has a positive effect by providing effective control over obesity. The results of our meta-analysis are consistent with previous results. Specifically, the average weight decreased by 8.66 kg (95% CI: −11.40 to −5.92), waist circumference reduced by 9.17 cm (95% CI: −10.67 to −7.66) and BMI reduced by 3.13 kg/m^2^ (95% CI: −3.31 to −2.95), as shown in Fig. 4.

## Discussion

The American Diabetes Society (ADA) recommended physical activity, dietary management, and medical intake and other approaches should be applied simultaneously to manage blood glucose levels, and other abnormal metabolic factors. KD showed numerous health benefits to patients with T2DM^22,23^. KD provides energy through fat oxidation. When the human body experienced extreme hunger or very limited carbohydrate, the ketone body was produced and released to circulation by hepatic transformation of fatty acids^24,25^. Nutritional ketosis is different from severe pathological diabetic ketosis; the blood ketone body remained at 0.5−3.0 mmol/L with reduced blood glucose and normal blood pH, with no symptoms in nutritional ketosis^26^.

The possible mechanism for the health benefit of KD on patients with T2DM is that the extreme restriction of carbohydrate reduces the intestinal absorption of mono-saccharide, which leads to lower blood glucose level and reduces the fluctuation of blood glucose, and its effectiveness on regulating glucose metabolism was confirmed by a large body of evidence^27,28^. The current study analyzed 13 studies from literature focusing on diabetic patients; the results showed that the reduction of blood glucose ranges from 0.62 to 5.61 mmol/L. Higher reduction amplitudes were reported by Dashti^29^ and Leonetti et al.^14^ of 5.61 mmol/L (weight random 3.0%) and 3.87 mmol/L (weight random 1.2%), respectively; other reductions in blood glucose were all lower than 1.8 mmol/L. The possible reason for the higher reduction found in these two studies could be the higher blood glucose level included in the studies, and also that the average blood glucose concentration was above 10.0 mmol/L, leading to the possibility of a larger reduction; however, their contribution to the overall effect estimations in the meta-analysis was low. The average changes in fasting blood glucose after the KD consumption among the selected studies were −1.29 mmol/L, indicating the effectiveness of the KD in lowering fasting blood glucose.

No studies included in this meta-analysis evaluated the effect of KD on postprandial glucose level; unlike medications, dietetic therapy showed a long-term effect on glucose regulation^4,16^, and HbA1c was analyzed to evaluate the long-term effect of KD. HbA1c effectively reflects the blood glucose control in the past 2−3 months in patients with diabetes. It is reported that the risk of cardiac infarction and micro-vascular complications reduced by 14% and 37%, respectively, when HbA1c reduced by 1%. Therefore, the HbA1c level showed essential clinical significance in evaluating the blood glucose control, revealing the potential problems in the treatment and thereby guiding the therapeutic schedule^30,31^. Eight of the selected studies showed a reduction of HbA1c after KD consumption, the changes ranging from −0.6% to −3.3%; HbA1c reduced <1.5% in the majority of the studies included in the current analysis besides the study conducted by Walton (−3.3%; weight random 5.1%)^15^. The possible explanation for such strong improvement of HbA1c could be that Walton’s study had enrolled a limited number of patients and thus the compliance of patients to KD therapy can be guaranteed. Moreover, the studied subjects were newly diagnosed diabetic patients who were under dietary management without taking glucose-lowering medications; newly diagnosed subjects persist well in the study. Considering the causal factors comprehensively, the above study showed an ideal reduction in HbA1c. The average reduction of HbA1c was 1.07 in the current analysis of the selected eight studies, indicating that dietary management may also achieve the ideal therapeutic effects of medication.

HOMA-IR is considered as an indicator to evaluate the status of insulin resistance. Insulin resistance as a clinical characteristic of T2DM is closely related to obesity. Improving insulin resistance is one of the major targets in diabetes treatment^32–34^. However, studies focusing on the role of KD in the improvement of insulin resistance in patients with diabetes are very limited; most of the studies focused on the effect in obese subjects^35,36^. For instance, a controlled clinical trial aiming at the effects of KD consumption in obese people without diabetes revealed that HOMA-IR decreased by about 2.0 after KD consumption for 6 weeks^37^. The current analysis showed consistent changes in the studies that included HOMA-IR evaluation, with reduction ranging from −0.4 to −3.4; the reason for the significant reduction of 3.4 in the study by Tay et al.^38^ is that the population included was obese diabetic patients with BMI higher than 30 kg/m^2^. Obesity is closely related to insulin resistance; KD consumption is confirmed to be effective in reducing body weight, and it is expected that KD may improve insulin resistance in obese diabetic patients^39^. During the ketogenesis, the sensitivity of the insulin receptor is promoted; therefore, KD not only ensures the supply of basic nutrients but also maintains a negative balance of energy, and reduces the fluctuation and reduction of insulin secretion caused by reduced carbohydrate intake as well, which eventually leads to improved insulin sensitivity^40–43^.

Consumption of KD not only improved glucose metabolism, but a large body of evidence has reported that KD improved lipid metabolism as well. Hussain et al.^4^ reported that KD reduced TG and TC, and increased HDL level, thus ameliorating the status of dyslipidemia. In the present study, eight studies included showed results of lipid metabolism in diabetic patients after KD consumption; however, only five analyzed the TC levels. The current results showed the mean reduction of TG was 0.72 mmol/L, TC was 0.33 mmol/L, and LDL was 0.05 mmol/L, while the increase of HDL was 0.14 mmol/L. The higher amplitude of variation occurred in the Dashti et al. study^29^. This study reported that TG reduced by 3.67 mmol/L, TC reduced by 1.88 mmol/L, and LDL reduced by 1.78 mmol/L, while HDL increased by 0.14 mmol/L. Changes in the amplitude of the lipid biomarkers were all at the higher end in the above study. Both glucose and lipid metabolism showed great improvement after KD consumption in such a study; the characteristics of subjects recruited were closely correlated. The study recruited 31 obese subjects with hyperglycemia, dyslipidemia, and BMI over 30 kg/m^2^. The baseline TG, TC, and LDL were higher than those of typical patients with T2DM, which may contribute to the significant changes after the intervention. Consumption of KD showed a significant therapeutic effect in common patients with T2DM, including the Dashti^29^ study. Disorders of lipid metabolism are particularly strong among patients with insulin resistance in T2DM. Dyslipidemia is lipotoxic to cells, leading to and/or aggravating insulin resistance. Its typical manifestation is the increase of TG and free fatty acid (FFA)^44–47^. Increased FFA is an independent pathogenic factor for insulin resistance and can possibly increase the risk for cardiovascular diseases^48,49^. Therefore, the improvement of dyslipidemia is beneficial for not only regulating insulin sensitivity but also controlling the occurrence and progression of diabetic complications^50,51^.

Numerous studies have confirmed the role of KD consumption in weight reduction in obese patients^35–37,40–43,52^; the current meta-analysis focused on the effect of KD on weight reduction in obese diabetic patients. The results showed the average reduction of body weight was 8.66 kg, waist circumference was 9.17 cm, and BMI was 3.22 kg/m^2^, which were consistent with previous studies in nondiabetic patients. We also found that KD reduced systolic blood pressure by 4.30 (95% CI: −7.02 to 1.58) and diastolic blood pressure by 5.14 (95% CI: −10.18 to 0.10) in patients with T2DM, which possibly benefit from the improvement of body weight^51^.

Besides the mediation of glucose and lipid metabolism, KD may also benefit other clinical symptoms in diabetic patients, including insomnia, chills, constipation, pruritus, numbness of limbs, hypopsia, fatty liver, hypertension, and reduced cardiac function.

The potential side effects of KD were only mentioned in two of the studies^14,41^ included in the meta-analysis; thus it is impossible to perform a systematic review in terms of the risks associated with KD consumption. Specifically, Goday and Leonetti’s^14,41^ study investigated the adverse reactions of KD. Goday et al.^41^ mentioned that fatigue, headache, nausea and vomiting were more common in the KD diet group after a 2-week intervention, while constipation and orthostatic hypotension were more common after 10 weeks. It was revealed by Leonetti et al.^14^ that in the early stages of applying the KD, patients reported a sense of hunger, but it could be significantly alleviated with the progress of the intervention. Even though headache, nausea, vomiting, constipation, diarrhea, and other symptoms were reported during the study, the symptoms were mild and lasted for a short time, not relating to clinical practice.

### Limitations

Only 13 studies were included in the current analysis, with limited studies focusing on the effect of KD in patients with T2DM worldwide. For instance, no analysis was conducted on HOMA-IR even though there was a trend of improvement; also, very limited literature was available. All studies included in this meta-analysis were carried out among Caucasian diabetic patients (no East Asians included); however, the majority of East Asian diabetic patients showed insulin resistance with central obesity and defect in insulin secretion. Therefore, clinical trials conducted among East Asians are highly desirable to confirm whether there is an improvement in the secretion function of islet cells other than improved regulation of glucose and lipid metabolism. Moreover, the current study analyzed the data without assigning studies into time duration due to the limited number of studies and the missing data of insulin and lipid biomarkers; in addition, the duration of the follow-up was decentralized into days, months, and years. The available studies concerning the effects of ketogenic diet in patients with diabetes are very limited; it is impossible to summarize a similar follow-up interval for statistical analysis of time points. However, the current results suggested that ketogenic diet consumption contributed to therapeutic effects despite the length of the term of intervention. The analysis of the difference before and after the intervention may also give credit to the clinical efficacy of the diet therapy. In current clinical practice, a majority of the patients have to use a combination of multiple drugs to improve their glycolipid metabolism. Drug therapy is a heavy mental and economical burden to patients. Given the fact that most of the patients are confused regarding a proper dietary therapy plan, it is essential to recommend a feasible dietary therapy plan to transmit a positive message to both patients with diabetes and physicians majored in the area of diabetes.

## Conclusion

Based on a meta-analysis that systematically reviewed 13 relevant studies, we found that ketogenic diet can not only control fasting blood glucose and reduce glycosylated hemoglobin, but also improve lipid metabolism. Additionally, ketogenic diet can reduce BMI and body weight. Therefore, ketogenic diet may be used as part of the integrated management of type 2 diabetes.
